# A voluntary use of insecticide treated nets can stop the vector transmission of Chagas disease

**DOI:** 10.1371/journal.pntd.0008833

**Published:** 2020-11-03

**Authors:** Cheol Yong Han, Habeeb Issa, Jan Rychtář, Dewey Taylor, Nancy Umana

**Affiliations:** 1 Department of Mechanical and Nuclear Engineering, Virginia Commonwealth University, Richmond, Virginia, USA; 2 Department of Biology, Virginia Commonwealth University, Richmond, Virginia, USA; 3 Department of Mathematics and Applied Mathematics, Virginia Commonwealth University, Richmond, Virginia, USA; 4 Department of Psychology, Virginia Commonwealth University, Richmond, Virginia, USA; UMR CNRS–IRD 2724, FRANCE

## Abstract

One of the stated goals of the London Declaration on Neglected Tropical Diseases is the interruption of domiciliary transmissions of Chagas disease in the region of the Americas. We used a game-theoretic approach to assess the voluntary use of insecticide treated nets (ITNs) in the prevention of the spread of infection through vector bites. Our results show that individuals behave rationally and weigh the risks of insect bites against the cost of the ITNs. The optimal voluntary use of ITNs results in predicted incidence rates that closely track the real incidence rates in Latin America. This means that ITNs are effective and could be used to control the spread of the disease by relying on individual decisions rather than centralized policies. Our model shows that to completely eradicate the vector transmission through the voluntary individual use of ITNs, the cost of ITNs should be as low as possible.

## Authors summary

We construct a game-theoretic model of individual use of insecticide treated nets (ITNs) to prevent the vector transmission of Chagas disease within the household.

Our results show that individuals behave rationally and weigh the risks of insect bites against the cost of the ITNs. The optimal voluntary use of ITNs results in predicted incidence rates that closely track the incidence rates in Latin America.

This means that ITNs are effective and could be used to control the spread of the disease by relying on individual decisions rather than centralized policies.

## Introduction

American trypanosomiasis, in humans known as Chagas disease, is one of the world’s most important neglected tropical diseases [1] and is most prevalent in Latin America where it is endemic in all 33 countries [2]. One of the stated goals of the London Declaration on Neglected Tropical Diseases is the interruption of domiciliary transmissions in the region of the Americas [3]. Worldwide 6–7 million people had been diagnosed with Chagas disease [4], and the actual numbers may be even higher [5]. There is a lack of research on Chagas disease despite the fact that (1) there are 50,000 to 200,000 new infections per year [6], (2) the disease results in an estimated $627.46 million in healthcare costs and $7.19 billion in societal costs annually [7], and (3) Chagas disease is still the largest parasitic disease burden on the American continent [8].

Chagas disease is a parasitic infection caused by the protozoan *Trypanosoma cruzi* [9]. There are two main forms of transmission: blood transfusion, most common in urban areas, and vector biting, most common in rural areas [10]. Chagas disease can also be contracted through organ transplantation, ingestion of parasite-contaminated food, and from mother to fetus [11, 12]. Three species of *Triatominae* insects, nicknamed kissing bugs, are the most common vectors. *T. infestans* is the primary vector in sub-Amazonian endemic regions (southern South America), *Rhodnius prolixus* is typically reported in northern South America and Central America, and *T. dimidiata* occupies a similar area, but also extends further north into Mexico [13].

Historically, the control of Chagas disease has focused on vector reduction using insecticides or indirect vector control through housing modifications [1, 14]. Still, preventive measures for vector transmission have remained a challenge due to the disease being closely linked to poverty and poor housing infrastructure such as mud and thatched houses that lack sanitary supervision [6]. Currently, no vaccine has been created in order to help stop the spread of Chagas disease [15]. Preventative measures in high risk areas include (1) avoiding sleeping in mud and thatched housing, (2) using insecticide treated netting (ITN) over one’s bed, and (3) using insect repellent on exposed skin [11, 16, 17]. Even in relatively poor areas, the use of ITNs was already demonstrated to be very cost-effective [18]. However, human behavior such as inconsistent use due to hot weather or inadequate education diminishes the effectiveness of ITNs [19].

From the behavioral perspective, disease prevention, such as ITNs use, produces public goods (herd immunity) that is non-rivalrous and non-exclusive [20]. Individuals often act in a way that maximizes their self-interests rather than the interests of the entire group [21, 22]. Disease prevention is thus prone to free-riding. The “free-riders” avoid the costs associated with the use of ITNs while they benefit from the preventive actions of others. This social dilemma is captured by the game theory framework [23]. The framework has now been applied to help model the prevention of many diseases such as African trypanosomiases [24], chikungunya [25], cholera [26], dengue [27], ebola [28], hepatitis B [29], hepatitis C [30], meningitis [31], monkeypox [32], polio [33], toxoplasmosis [34], typhoid [35] and many others, see for example [36] and [22] for recent reviews.

There are many mathematical models of Chagas disease [37], including [1, 10, 11, 38–43]. In this paper, we consider a recent, yet relatively simple model of Chagas disease dynamics developed in [44]. This model includes vector transmission by *Rhodnius prolixus* as well as the interplay of palm plantations and human settlements (domestic) habitats for the vectors. We consider the use of ITNs as a mode of voluntary protection from vector bites, similarly to what was done in [45] for malaria. We apply a game-theoretic approach to evaluate individual and population-wide use of ITNs. We also perform the sensitivity analysis and validate the model on Chagas disease incidence data.

## Mathematical model of Chagas disease dynamics

**Fig 1:**
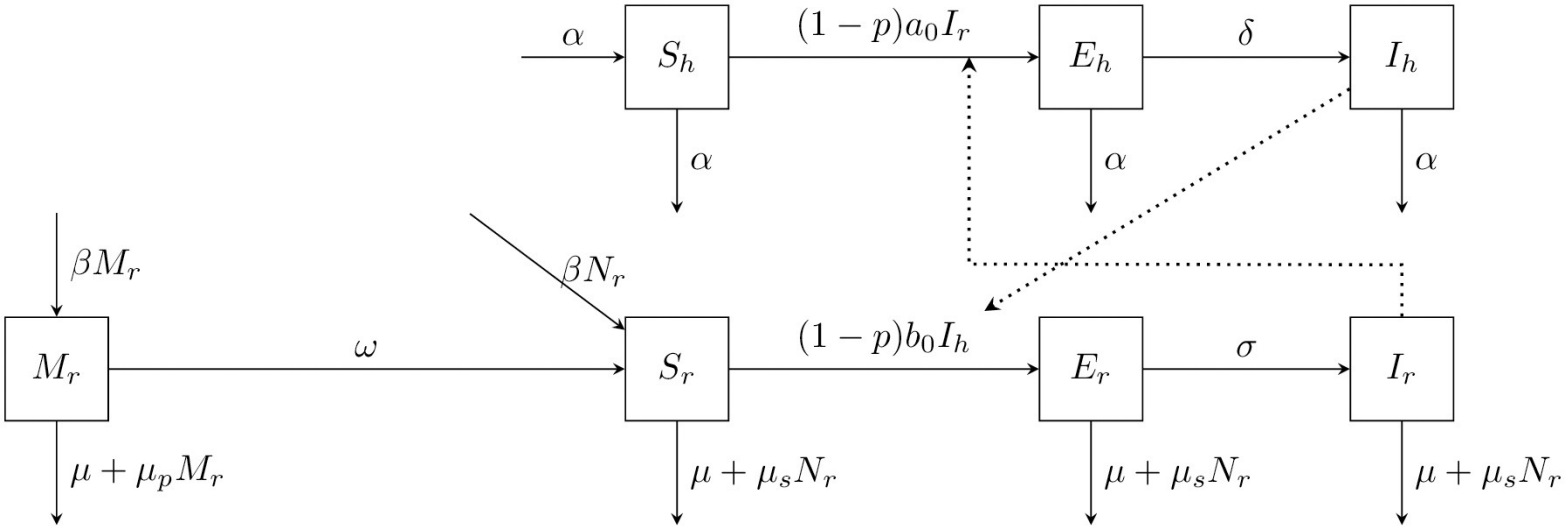
The scheme of Chagas disease population dynamics in humans and vectors *R. prolixus*, adapted from [44]. Human population is at the top, *R. prolixus* at the bottom, the *M*_*r*_ compartment represents vectors in the palm plantation, the remaining compartments represent populations in the settlement. Solid lines represent movement to and from the compartments, dotted lines demonstrate the influence on the transmission rates.

We extend the compartmental epidemiological model of Chagas disease introduced in [44] by incorporating the use of ITNs. The model, shown in Fig 1, describes the interactions between the vectors, *Rhodnius prolixus*, and the host (human) populations denoted by *r* and *h* subscripts, respectively. The model involves two separate areas: the palm plantation and human settlement. The palm plantation acts as a potential reservoir of vectors. Since *R. prolixus* usually bites humans at night [46], the transmission is modelled only through contact between vectors and humans in the settlement. For simplicity, we do not consider any infected vectors in the plantation area, although infected *R. prolixus* can be found in the palm trees as well [47]. We also do not consider any human dynamics in the plantation area.

There is no recovery from Chagas disease [16] so the total human population is divided into (1) susceptible (*S*_*h*_), i.e. not infected with *T. cruzi*, (2) exposed (*E*_*h*_), i.e. infected with *T. cruzi* but not yet infectious, and (3) infectious (*I*_*h*_). The population size is normalized so that *N*_*h*_ = *S*_*h*_ + *E*_*h*_ + *I*_*h*_ = 1. The vector population is divided into vectors living in the plantation (*M*_*r*_) and the settlement (*N*_*r*_). The population in the plantation is always susceptible. The vector population in the settlement is divided into susceptible (*S*_*r*_), exposed (*E*_*r*_) and infectious (*I*_*r*_).

Humans are born as susceptible at the per capita rate *α*. While there is a disease induced mortality [48], we follow [44] in omitting this for the sake of simplicity of the model. To keep the human population constant, the human natural death rate is considered the same as the birth rate and the same across all classes. The vectors are born as susceptible at per capita rate *β*; the birth rate is considered the same in the plantation and the settlement. The vectors follow a logistic growth with carrying capacity *K*_*p*_ in the plantation and *K*_*s*_ in the settlement. The vectors migrate from the plantation to the settlement at rate *ω*. The natural death rate of vectors, *μ*, is considered the same across all classes. We assume *μ* < *β*.

The disease is transmitted from an infectious vector to susceptible humans at rate (1 − *p*)*a*_0_ and from an infectious human to susceptible vectors at rate (1 − *p*)*b*_0_. Here, *p* is the frequency of ITN use while *a*_0_ and *b*_0_ are the transmission rates without ITN use. After the incubation period, the exposed individuals become infectious at rate *δ* for humans and *σ* for vectors.

The values and ranges of model parameters are summarized in Table 1, details are presented below.

**Table 1:** Model parameters of Chagas disease dynamics. Detailed estimates of parameter values and ranges are presented in the text. All rates are per capita per day. The costs are in USD per year. The carrying capacities are in the number of vectors per person.

Symbol	Meaning	Base value	Reference(s)
*a*_0_	Vector-to-host transmission rate	1.74 × 10^−4^	[49]
*b*_0_	Host-to-vector transmission rate	19 × 10^−4^	[50]
*p*	Frequency of ITNs use	variable in [0, 1]	
*α*	Host recruitment and death rate	1/(75 × 365)	[51]
*β*	Birth rate of vectors	0.022	[52]
*μ*	Natural death rate of vectors	0.008	[52]
*δ*	Incubation rate in humans	0.1	[13]
*σ*	Incubation rate in vectors	0.143	[53]
*K*_*s*_	Carrying capacity of vectors in settlement	10	[54]
*K*_*p*_	Carrying capacity of vectors in plantation	200	[55]
*μ*_*s*_	Density-dependent death rate of vectors in the settlement	$\frac{\beta -\mu }{K_{s}}$	
*μ*_*p*_	Density-dependent death rate of vectors in the plantation	$\frac{\beta -\mu }{K_{p}}$	
μ*	Mortality rate of vectors in settlement in equilibrium	*μ* + *μ*_*s*_ *N*_*r*_	
*ω*	Migration rate of vectors	0.01	[56]
*C*_ITN_	Cost of ITN use	2.5	[57, 58]
*C*_Chagas_	Cost of Chagas infection	383	[7]

### Parameter estimation

The vector-to-human transmission rate, *a*_0_, is estimated as 5.8 × 10^−4^ ⋅ 0.3, i.e. 1.74 × 10^−4^ per day with range [5.2, 44] × 10^−5^ by the following reasoning. Using various datasets from literature, [49] estimated the probability of vector-to-human transmission (per contact) as 5.8 × 10^−4^ (with 95% CI: [2.6, 11.0] × 10^−4^). This estimate is consistent across triatomine species, robust to variations in other parameters, and corresponds to 900–4,000 contacts per case. Moreover, [59] estimates the biting rate to be between 0.2 − 0.4 and [60] and [49] further confirm this by stating that the biting rate on average is 0.3 per day. We note that [61] found feeding rate of *T. infestants* to be between 0.3 and 0.6 per day.

Similarly, from the data presented in [50, Table 4], we estimate the host-to-vector transmission rate, *b*_0_, as 0.0062 ⋅ 0.3 = 19 × 10^−4^ per day with the range [3.8, 32] × 10^−4^ per day.

The average life expectancy age in Latin America is 75 years, ranging from 63 in Haiti to 80 in Chile and Costa Rica [51]. This gives the average rate (75 ⋅ 365)^−1^ = 3.65 × 10^−5^ per day with the range [3.42, 4.35] × 10^−5^ per day.

The average instantaneous birth rate, *β* is given as 0.158 per week with a range [0.120, 0.208] per week in [52, Table 7]. This yields the average 0.022 per day with the range [0.017, 0.30] per day.

The life expectancy of various Triatoma species ranges from 73 days to 268 days [62]. This gives the range for the vector death rate as [0.0037, 0.0136] per day. Further estimates for instantaneous death rate of *T. infestants* ranging from 0.023–0.109 per week are given in [52, Table 7]. This yields an average $\frac{0.056}{7}=0.008$ per day and the range [0.0033, 0.016] per day. We note that [63] assume the death rate (for *T. infestants*) to be 0.0046 per day with a reference to [52]; however we believe that this discrepancy is likely caused by [63] considering the standard deviation 0.034 instead of the average rate 0.056 per week in [52]. Also, [1] uses the death rate 1.73 per year (i.e. 0.0047 per day) with a reference to [62]. For the purpose of our study we will use 0.008 per day as the average death rate with the range [0.0033, 0.016] per day.

The incubation period for humans after exposure to a triatomine bite is 5–14 days [13]. As noted by [12], definitive data is not available because persons who live in areas of active transmission are generally continually at risk for exposure to the vectors. We assume that the incubation is 10 days on average. Therefore, the incubation rate in humans, *δ*, was estimated as 0.1 per day on average with a range can be represented by [0.071, 0.2] per day.

The average incubation rate in vectors, *σ*, is estimated as 1/7 per day with the range [1/8, 1/6] per day. This is based on [53] who found that 3–4 days post-infection, the *T. cruzi* parasite population begins to colonize the triatomine hosts to reach a climax at day 7 post-infection, which is maintained during the next two weeks.

Our model is normalized with a total human population equal to 1, and we use *K*_*s*_ = 10, i.e. 10 bugs per person as used in [1]. We note that [54] use 10, 50, 100 and cite variable bug numbers in studies [64–67]. Other study, [43], uses 158 bugs per household [68].

The carrying capacity of vectors in the field is 31, 900 per km^2^ [55]. The small plantation size is about 5ha [69], yielding 1600 bugs, i.e. after renormalization for a 8 person household, we get *K*_*p*_ = 200.

The migration rate, *ω*, was set to 0.01 per day on average with the range [0, 0.02] [56]. We note that [56] cites [70] as the source for that number, but we were not able to locate the information in [70]. Also, [44] and [2] use *ω* = 0.05. However, for the other values of the parameters, most notably the vector birth rate *β* and death rate *μ* such a high value of *ω* would result in no bugs in the plantation area. Consequently, we adapted the value 0.01 from [56]. Moreover, as seen from the sensitivity analysis, the results are not overly sensitive to values of *ω*.

## Analysis

The model of the transmission dynamics shown in Fig 1 yields the following system of differential equations.

$$\begin{array}{cc} \frac{dS_{h}}{dt} & =\alpha -\alpha S_{h}-\left(1-p\right)a_{0}I_{r}S_{h} \end{array}$$ (1)

$$\begin{array}{cc} \frac{dE_{h}}{dt} & =\left(1-p\right)a_{0}I_{r}S_{h}-\left(\alpha +\delta \right)E_{h} \end{array}$$ (2)

$$\begin{array}{cc} \frac{dI_{h}}{dt} & =\delta E_{h}-\alpha I_{h} \end{array}$$ (3)

$$\begin{array}{cc} \frac{dM_{r}}{dt} & =\left(\beta -\mu \right)M_{r}(1-\frac{M_{r}}{K_{p}})-\omega M_{r} \end{array}$$ (4)

$$\begin{array}{cc} \frac{dS_{r}}{dt} & =\omega M_{r}+\beta N_{r}-(\left(1-p\right)b_{0}I_{h}+\mu +\mu_{s}N_{r})S_{r} \end{array}$$ (5)

$$\begin{array}{cc} \frac{dE_{r}}{dt} & =\left(1-p\right)b_{0}I_{h}S_{r}-(\mu +\sigma +\mu_{s}N_{r})E_{r} \end{array}$$ (6)

$$\begin{array}{cc} \frac{dI_{r}}{dt} & =\sigma E_{r}-(\mu +\mu_{s}N_{r})I_{r} \end{array}$$ (7)

### Equilibria of the ODE system (1)–(7)

There are four possible equilibria of the dynamics (1)–(7): (i) an unstable disease-free equilibrium $E_{0}^{1}=(1,0,0,0,0,0,0)$, (ii) a disease-free equilibrium $E_{0}^{2}=(1,0,0,0,K_{s},0,0)$, (iii) a disease-free equilibrium $E_{0}^{3}=(1,0,0,M_{r}^{*},N_{r}^{*},0,0)$ where

$$\begin{array}{cc} M_{r}^{*} & =K_{p}\frac{\beta -\mu -\omega }{\beta -\mu }, \end{array}$$ (8)

$$\begin{array}{cc} N_{r}^{*} & =K_{s}\frac{1+\sqrt{1+4\frac{\omega M_{r}^{*}}{K_{s}\left(\beta -\mu \right)}}}{2}, \end{array}$$ (9)

and finally (iv) the endemic equilibrium $E^{*}=(S_{h}^{*},E_{h}^{*},I_{h}^{*},M_{r}^{*},S_{r}^{*},E_{r}^{*},I_{r}^{*})$ where

$$\begin{array}{cc} E_{h}^{*} & =\frac{N_{r}^{*}\left(1-p\right)a_{0}-\frac{\mu^{*}}{\sigma }\frac{\mu^{*}+\sigma }{\left(1-p\right)b_{0}\frac{\delta }{\alpha }}\left(\alpha +\delta \right)}{\left(\alpha +\delta \right)(1+\frac{\mu^{*}}{\sigma }+N_{r}^{*}\frac{\left(1-p\right)a_{0}}{\alpha })} \end{array}$$ (10)

$$\begin{array}{cc} I_{h}^{*} & =\frac{\delta }{\alpha }E_{h}^{*} \end{array}$$ (11)

$$\begin{array}{cc} S_{h}^{*} & =1-E_{h}^{*}-\frac{\delta }{\alpha }E_{h}^{*} \end{array}$$ (12)

$$\begin{array}{cc} I_{r}^{*} & =\frac{E_{h}^{*}\left(\alpha +\delta \right)}{\left(1-p\right)a_{0}(1-E_{h}^{*}(1+\frac{\delta }{\alpha }))} \end{array}$$ (13)

$$\begin{array}{cc} E_{r}^{*} & =\frac{\mu^{*}}{\sigma }I_{r}^{*} \end{array}$$ (14)

$$\begin{array}{cc} S_{r}^{*} & =\frac{\sigma +\mu^{*}}{\left(1-p\right)b_{0}I_{h}^{*}}(I_{r}^{*}\frac{\mu^{*}}{\sigma }) \end{array}$$ (15)

and $\mu^{*}=\mu +\mu_{s}N_{r}^{*}$ denotes the vector mortality rate in the settlement when the population levels reach the equilibrium. See the section below for detailed calculations.

### Step-by-step calculations for equilibria

Let us set *N*_*r*_ = *S*_*r*_ + *E*_*r*_ + *I*_*r*_ and investigate the system

$$\begin{array}{cc} \frac{dM_{r}}{dt} & =\left(\beta -\mu \right)M_{r}(1-\frac{M_{r}}{K_{p}})-\omega M_{r} \end{array}$$ (16)

$$\begin{array}{cc} \frac{dN_{r}}{dt} & =\left(\beta -\mu \right)N_{r}(1-\frac{N_{r}}{K_{s}})+\omega M_{r} \end{array}$$ (17)

that results from (4) and from the sum of eqs (5) and (6), and (7).

The system (16) and (17) has three equilibria: (a) (0, 0) which is always unstable (if *β* > *μ*), (b) (0, *K*_*s*_) which is locally stable if *β* − *μ* < *ω*, and finally (c) $\left(M_{r}^{*},N_{r}^{*}\right)$ where

$$\begin{array}{cc} M_{r}^{*} & =K_{p}\frac{\beta -\mu -\omega }{\beta -\mu }, \end{array}$$ (18)

$$\begin{array}{cc} N_{r}^{*} & =K_{s}\frac{1+\sqrt{1+4\frac{\omega M_{r}^{*}}{K_{s}\left(\beta -\mu \right)}}}{2}, \end{array}$$ (19)

which is defined and locally stable if *ω* < *β* − *μ*.

Now, let us proceed to solve for the equilibria of the system (1)–(7). Based on above calculations, we have to solve the following system of algebraic equations

$$\begin{array}{cc} 0 & =\alpha \left(1-S_{h}\right)-\left(1-p\right)a_{0}S_{h}I_{r} \end{array}$$ (20)

$$\begin{array}{cc} 0 & =\left(1-p\right)a_{0}S_{h}I_{r}-E_{h}\left(\alpha +\delta \right) \end{array}$$ (21)

$$\begin{array}{cc} 0 & =\delta E_{h}-\alpha I_{h} \end{array}$$ (22)

$$\begin{array}{cc} 0 & =\omega M_{r}^{*}+\beta N_{r}^{*}-S_{r}(\left(1-p\right)b_{0}I_{h}+\mu +\mu_{s}N_{r}^{*}) \end{array}$$ (23)

$$\begin{array}{cc} 0 & =\left(1-p\right)b_{0}S_{r}I_{h}-E_{r}(\mu +\sigma +\mu_{s}N_{r}^{*}) \end{array}$$ (24)

$$\begin{array}{cc} 0 & =\sigma E_{r}-I_{r}(\mu +\mu_{s}N_{r}^{*}) \end{array}$$ (25)

where $M_{r}^{*}$ is given by (18) and $N_{r}^{*}$ is given by (19). Let us set $\mu^{*}=\mu +\mu_{s}N_{r}^{*}$.

By adding (20),(21), and (22), we get

$$\begin{array}{cc} N_{h} & =S_{h}+I_{h}+E_{h}=1. \end{array}$$ (26)

We have the disease-free equilibrium with *S*_*h*_ = 1, *E*_*h*_ = 0, *I*_*h*_ = 0, *S*_*r*_ = *N*_*r*_, *E*_*r*_ = 0, *I*_*r*_ = 0 whenever any of the following happens: (1) *I*_*r*_ = 0 (in particular when $N_{r}^{*}=0$), or (2) *I*_*h*_ = 0, or (3) *p* = 1.

For the rest of the section, we will assume that *p* < 1, *I*_*h*_ > 0 and *I*_*r*_ > 0. By (22),

$$\begin{array}{c} I_{h}=\frac{\delta E_{h}}{\alpha } \end{array}$$ (27)

and so by (26) and (27),

$$\begin{array}{c} S_{h}=1-E_{h}-\frac{\delta }{\alpha }E_{h}. \end{array}$$ (28)

By (25),

$$\begin{array}{c} E_{r}=I_{r}\frac{\mu^{*}}{\sigma } \end{array}$$ (29)

Using Eq (24)

$$\begin{array}{c} S_{r}=\frac{E_{r}\left(\sigma +\mu^{*}\right)}{\left(1-p\right)b_{0}I_{h}}=(\frac{\sigma +\mu^{*}}{\left(1-p\right)b_{0}I_{h}})(\frac{I_{r}\mu^{*}}{\sigma }) \end{array}$$ (30)

By (20),

$$\begin{array}{cc} I_{r} & =\frac{\alpha (1-S_{h})}{\left(1-p\right)a_{0}S_{h}}=\frac{\alpha (I_{h}+E_{h})}{\left(1-p\right)a_{0}S_{h}} \end{array}$$ (31)

$$\begin{array}{c} =\frac{E_{h}\left(\alpha +\delta \right)}{\left(1-p\right)a_{0}(1-E_{h}(1+\frac{\delta }{\alpha }))} \end{array}$$ (32)

Finally,

$$\begin{array}{cc} N_{r} & =S_{r}+I_{r}+E_{r} \end{array}$$ (33)

$$\begin{array}{cc} & =\frac{\mu^{*}+\sigma }{\left(1-p\right)b_{0}I_{h}}\frac{\mu^{*}}{\sigma }I_{r}+I_{r}+\frac{\mu^{*}}{\sigma }I_{r} \end{array}$$ (34)

$$\begin{array}{cc} & =I_{r}(1+\frac{\mu^{*}}{\sigma }+\frac{\mu^{*}+\sigma }{\left(1-p\right)b_{0}I_{h}}\frac{\mu^{*}}{\sigma }) \end{array}$$ (35)

$$\begin{array}{cc} & =E_{h}\frac{\alpha +\delta }{\left(1-p\right)a_{0}(1-E_{h}(1+\frac{\delta }{\alpha }))}(1+\frac{\mu^{*}}{\sigma }+\frac{\mu^{*}+\sigma }{\left(1-p\right)b_{0}\frac{\delta }{\alpha }E_{h}}\frac{\mu^{*}}{\sigma }) \end{array}$$ (36)

and thus

$$\begin{array}{c} E_{h}^{*}=\frac{N_{r}\left(1-p\right)a_{0}-\frac{\mu^{*}}{\sigma }\frac{\mu^{*}+\sigma }{\left(1-p\right)b_{0}\frac{\delta }{\alpha }}\left(\alpha +\delta \right)}{\left(\alpha +\delta )(1+\frac{\mu^{*}}{\sigma }+N_{r}\frac{\left(1-p\right)a_{0}}{\alpha }\right)} \end{array}$$ (37)

and the remaining values are given by

$$\begin{array}{cc} I_{h}^{*} & =\frac{\delta }{\alpha }E_{h}^{*} \end{array}$$ (38)

$$\begin{array}{cc} S_{h}^{*} & =1-E_{h}^{*}-\frac{\delta }{\alpha }E_{h}^{*} \end{array}$$ (39)

$$\begin{array}{cc} I_{r}^{*} & =\frac{E_{h}^{*}\left(\alpha +\delta \right)}{\left(1-p\right)a_{0}(1-E_{h}^{*}(1+\frac{\delta }{\alpha }))} \end{array}$$ (40)

$$\begin{array}{cc} E_{r}^{*} & =I_{r}^{*}\frac{\mu^{*}}{\sigma } \end{array}$$ (41)

$$\begin{array}{cc} S_{r}^{*} & =\frac{\sigma +\mu^{*}}{\left(1-p\right)b_{0}I_{h}^{*}}(I_{r}^{*}\frac{\mu^{*}}{\sigma }). \end{array}$$ (42)

### The basic reproduction number

The basic reproduction number, i.e. the number of secondary infections caused by a single infectious individual in an otherwise disease-free population is given by

$$\begin{array}{cc} R_{0} & ={\left(1-p\right)}^{2}R_{0}^{noITN} \end{array}$$ (43)

where

$$\begin{array}{c} R_{0}^{noITN}=(\frac{1}{\alpha })(b_{0}N_{r}^{*})(\frac{\sigma }{\sigma +\mu^{*}})(\frac{1}{\mu^{*}})(a_{0})(\frac{\delta }{\delta +\alpha }) \end{array}$$ (44)

is the number of secondary infections caused by a single infectious individual if nobody uses ITN. The formula can be derived as follows. An infectious individual lives on average for a time *α*^−1^. During that time, they expose susceptible vectors at the rate $(1-p)b_{0}N_{r}^{*}$. Each of the exposed vectors become infectious with probability $\frac{\sigma }{\sigma +\mu^{*}}$. Each of the infectious vectors then lives for the time μ*^−1^ during which it exposes susceptible individuals at the rate (1 − *p*)*a*_0_ *N*_*h*_ = (1 − *p*)*a*_0_. Each of those exposed individuals will become infectious with probability $\frac{\delta }{\delta +\alpha }$.

The equilibrium $E_{0}^{1}$ is always unstable. The disease-free equilibrium $E_{0}^{2}$ is locally stable if *ω* > *β* − *μ* and *R*_0_ < 1. The disease-free equilibrium $E_{0}^{3}$ exists if *ω* < *β* − *μ* and is stable if *R*_0_ < 1. The endemic equilibrium $E^{*}$ is defined and locally stable if *ω* < *β* − *μ* and ${ℛ}_{0}>1$.

### Herd immunity

It follows from (43) that $\frac{\partial {ℛ}_{0}}{\partial p}<0$, i.e. ${ℛ}_{0}$ is decreasing in *p*. Consequently, the population will reach herd immunity at the smallest value of *p* ∈ [0, 1] for which ${ℛ}_{0}\leq 1$, i.e.

$$\begin{array}{cc} p_{\text{HI}} & =\max\{0,1-{\left(R_{0}^{noITN}\right)}^{-1/2}\}. \end{array}$$ (45)

## Game-theoretic model of ITN use

### Model setup

In this section, we set up and solve a game-theoretic model of individual ITN use decisions. We will assume that the system (1)–(7) is in an equilibrium. Individuals can either use or not use an ITN. We assume that all individuals are rational and act in their own self-interest [21]. As usual in vaccination games, see for example [35], individuals weigh the perceived cost of ITN use against the risks of infection. The risk of infection depends on the population-wide ITN use rate. This results in a public goods game in which individuals base their ITN use decision on the decisions of others.

For simplicity, we will consider only the actual monetary costs of ITN use, but we note that perceived costs could involve other factors such as possible discomfort associated with limited air circulation, and possible side effects of the insecticide [45]. The cost varies, it can be about $5 in Mexico and $9 in Columbia [58]. An ITN lasts about 2 years [57], so the annual cost is *C*_ITN_ = $2.50 in Mexico and *C*_ITN_ = $4.5 in Columbia. The annual cost of a *T. cruzi* infection, *C*_Chagas_, in Latin America is estimated as $383 with a range $207–$636 [7]. From the perspective of an individual, the expected cost of not using ITNs when the probability of ITN use in the overall population is *p*, denoted by *C*_noITN_(*p*), is given as a product of *C*_Chagas_ and the probability of getting infected (the probability of moving from the *S*_*h*_ compartment to the compartment *I*_*h*_),

$$\begin{array}{c} C_{\text{noITN}}\left(p\right)=\{\begin{array}{cc} C_{\text{Chagas}}\cdot (\frac{a_{0}I_{r}^{*}}{a_{0}I_{r}^{*}+\alpha })\cdot (\frac{\delta }{\delta +\alpha }), & p<p_{\text{HI}},\\ 0, & p\geq p_{\text{HI}}, \end{array} \end{array}$$ (46)

where the herd immunity level of ITN use, *p*_HI_, is given in (45).

### Dependence of *C*_noITN_ on *p*

**Fig 2:**
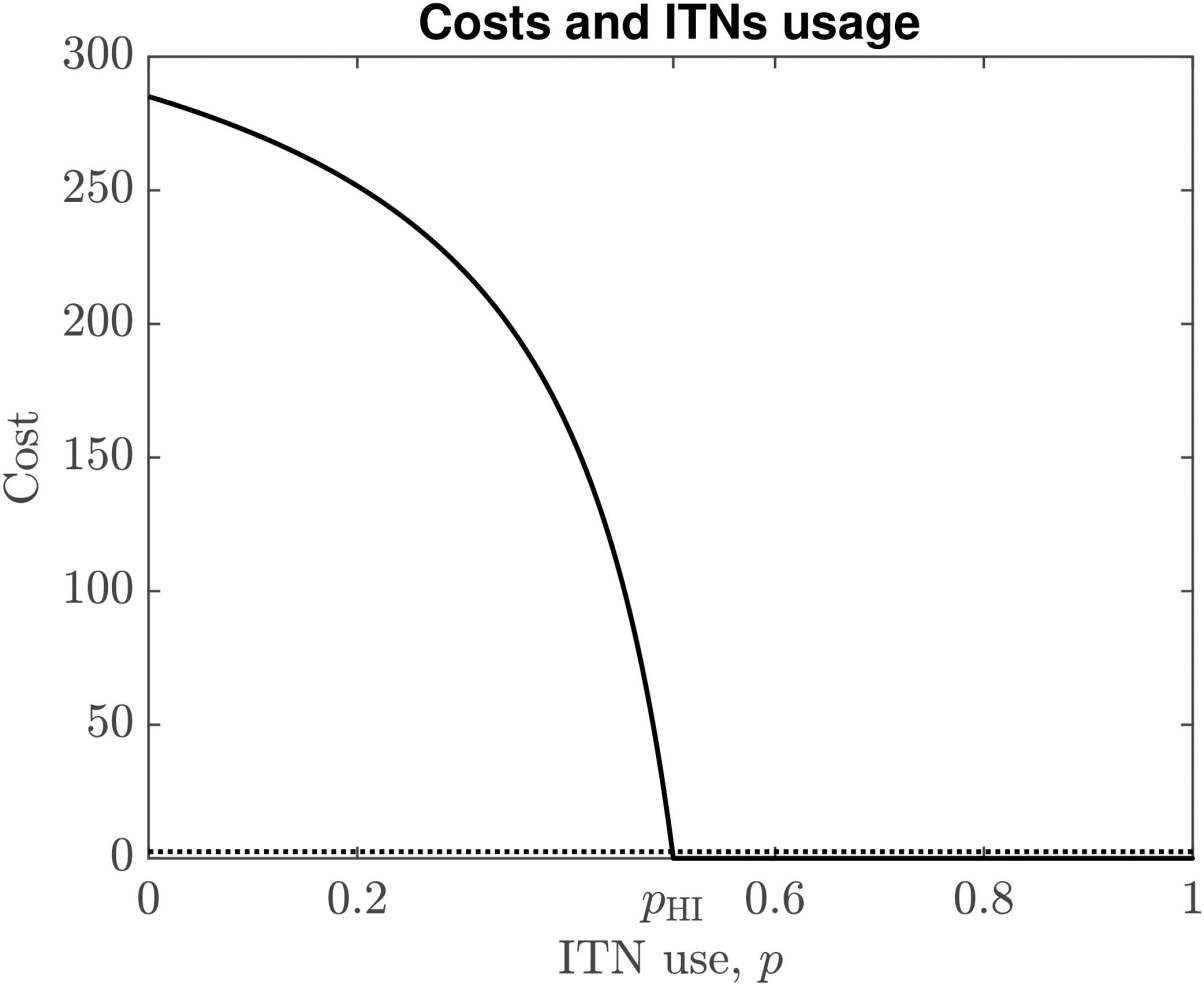
The expected cost of not using ITN (solid) as a function of ITNs use in the population. Parameters are as in Table 1. Herd immunity is achieved when the expected cost reaches 0. The Nash equilibrium ITN use is achieved at the intersection with the dotted line (the cost of the ITN). It follows that *p*_*NE*_ < *p*_HI_, but also *p*_*NE*_ ≈ *p*_HI_.

Here we show that *C*_noITN_(*p*) is decreasing in *p*; this is illustrated in Fig 2.

By (46) $\frac{\partial C_{noITN}}{\partial p}=\frac{\partial C_{noITN}}{\partial I_{r}^{*}}\cdot \frac{\partial I_{r}^{*}}{\partial p}$. Since $\frac{\partial C_{noITN}}{\partial I_{r}^{*}}>0$ and, as seen below, $\frac{\partial I_{r}^{*}}{\partial p}<0$, we will get that $\frac{\partial C_{noITN}}{\partial p}<0$. By (37),

$$\begin{array}{cc} E_{h}^{*} & =\frac{a_{0}}{\alpha +\delta }\cdot \frac{N_{r}\left(1-p\right)}{\left(1+\frac{\mu^{*}}{\sigma }+N_{r}\frac{\left(1-p\right)a_{0}}{\alpha }\right)}-\frac{\mu^{*}\left(\mu^{*}+\sigma \right)\alpha }{\sigma \left(1-p\right)b_{0}\delta \left(1+\frac{\mu^{*}}{\sigma }+N_{r}\frac{\left(1-p\right)a_{0}}{\alpha }\right)} \end{array}$$ (47)

and the first term is increasing in (1 − *p*) and thus decreasing in *p* while the second term is decreasing in (1 − *p*) and thus increasing in *p*. Thus, $\frac{\partial E_{h}^{*}}{\partial p}<0$.

Consequently, by (38), $\frac{\partial I_{h}^{*}}{\partial p}<0$. Now, for the contradiction, assume that $\frac{\partial I_{r}^{*}}{\partial p}>0$. By (41), $\frac{\partial E_{r}^{*}}{\partial p}>0$. Since $S_{r}^{*}=N_{r}^{*}-E_{r}^{*}-I_{r}^{*}$ and $\frac{\partial N_{r}^{*}}{\partial p}=0$, we get $\frac{\partial S_{r}^{*}}{\partial p}<0$. By (24), $E_{r}^{*}=\frac{(1-p)b_{o}S_{r}^{*}I_{h}^{*}}{\mu +\sigma +\mu_{s}N_{r}^{*}}$ and thus, as $S_{r}^{*}$ and $I_{h}^{*}$ are decreasing in *p*, $E_{r}^{*}$ should be decreasing in *p*, contradiction with an already established fact that $\frac{\partial E_{r}^{*}}{\partial p}>0$.

### Nash equilibria

When the ITN use *p* is such that *C*_noITN_(*p*) = *C*_ITN_, the ITN use is at Nash equilibrium, *p*_NE_; this means that no individual has an incentive to deviate from their current ITN usage. Because *C*_noITN_(*p*) is decreasing in *p*, *p*_NE_ is in fact a convergently stable Nash equilibrium which indicates that the population will evolve toward it, see [71]. When *p* < *p*_NE_, it is beneficial for the individual to use the ITN (the cost of using the ITN is smaller than the expected cost of infection); when *p* > *p*_NE_, it is beneficial for the individual not to use the ITN (the cost of the net is larger than the expected cost of infection).

### Results, sensitivity analysis and model validation

**Fig 3:**
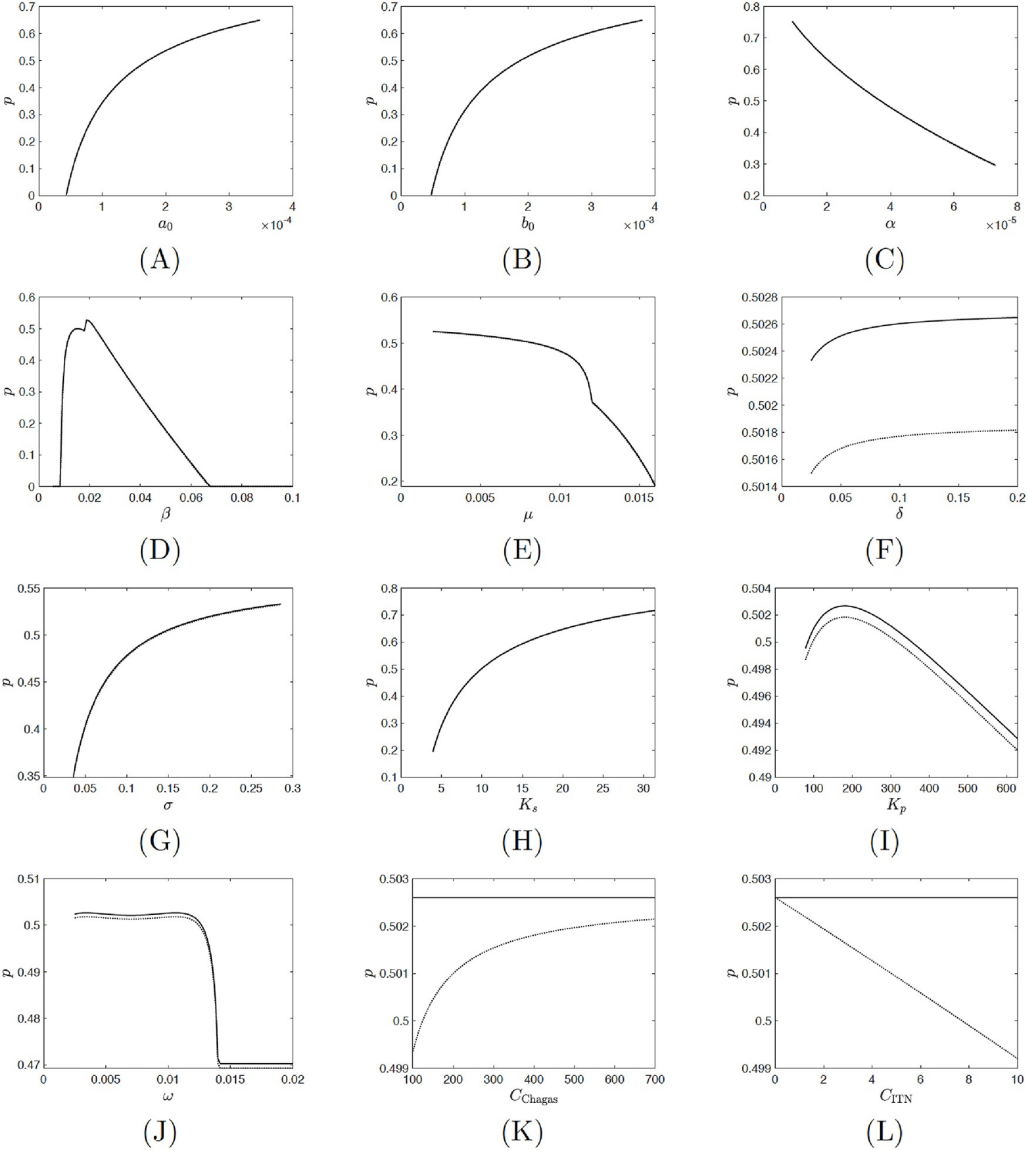
Dependence of *p*_HI_ (solid) and *p*_NE_ (dotted) on different parameter values. Unless varied, the parameter values are as specified in Table 1. For those parameters, *p*_HI_ = 0.5026 and *p*_NE_ = 0.5017.

The values of *p*_HI_ and *p*_NE_ are close together, see Figs 2 and 3. For the parameters as in Table 1, the model predicts *p*_HI_ = 0.5026 and *p*_NE_ = 0.5017.

More than 50% of the households in endemic areas of Colombia use bednets (although some were not insecticide treated) [73]. In Nicaragua, 34.2% households had bednets [19];the use of bednets is highest for the infants (45.8%) and decreases to 31.8% for children aged 1–4 years [19].

**Fig 4:**
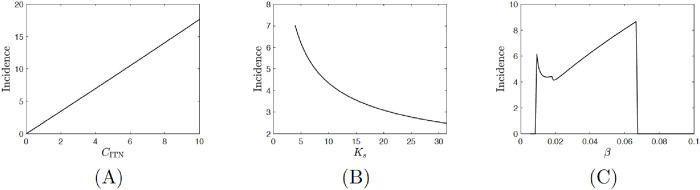
The incidence rate in person per year per 100,000 individuals when individuals use of ITN is optimal (at Nash equilibrium). Unless varied, the parameters are as in Table 1.

Our model predicts the annual incidence rate, the number of new infections per year, $i=(1-p)a_{0}I_{r}S_{h}\left(\frac{\delta }{\alpha +\delta }\right)$, to be about 4.72 person per year per 100,000 individuals when ITN use is at Nash equilibrium, see Fig 4. This agrees with the annual incidence rate due to vector transmission in Latin America and Mexico, both at 5 person per year per 100,000 individuals [74]. When we assume *C*_ITN_ = 4.5, to more closely match the price in Colombia, our model predicts incidence rate around 8.37 person per year per 100,000 individuals, again in close agreement with the real incidence rate of 11 person per year per 100,000 individuals [74]. This all indicates that individuals behave rationally as predicted in general by [21]. Our findings also agree with [75] whose results showed that peoples’ acceptance of ITN use is related to the perception of an immediate protective effect against vectors.

Our crucial result is that the incidence rate is essentially linear and increasing with the cost of the ITNs, see Fig 4A. It follows that to reduce the incidence of Chagas disease, one should reduce the cost *C*_ITN_ as much as possible.

Fig 4B shows how the incidence rates depend on the number of triatomines at home (*K*_*s*_). When *K*_*s*_ is small, the incidence rate is 0. Once *K*_*s*_ increases above a certain threshold, the incidence rate increases rapidly, but then it decreases in *K*_*s*_. This agrees with [39]; they showed that it is best to have no dogs in the household (low *K*_*s*_) but that once there are dogs in the house, human infection declines with the number of dogs (i.e. with increasing *K*_*s*_), allowing the dogs to sufficiently divert vectors away from the humans. In [11] they also conclude that reducing the population of triatomines and keeping domestic animals out of the households is the best way to decrease the risk of human infections.

Table 2 shows the sensitivity indices of *p*_HI_, *p*_NE_, the difference *p*_HI_ − *p*_NE_ and the incidence *i* on model parameters. Since *p*_NE_ and *p*_HI_ are very close to each other, their sensitivity indices are almost identical. We note that, if we disregard human birth rate, *α*, that cannot be easily individually adjusted, the herd immunity level *p*_HI_ is most sensitive to the vector birth rate *β*, settlement carrying capacity *K*_*s*_ and the transmission rates *a*_0_ and *b*_0_. In all cases, the sensitivity index is about 0.5 (or − 0.5 in the case of *β*), meaning that 1% increase of the parameter causes *p*_HI_ to increase (decrease in the case of *β*) by about 0.5%. Since *p*_NE_ increases (decreases) slightly more than *p*_HI_, the sensitivity index of *p*_HI_ − *p*_NE_ and of the incidence rate have reverse signs. The incidence rate is most sensitive to the cost of the ITNs, *C*_ITN_, and the vector birth rate, *β*. A decrease of the ITN cost causes the incidence rate to decrease. The dependence of *β* is more complex. The disease is endemic only for medium values of *β* ∈ (0.009, 0.068); there are not enough vectors for low *β* (in fact no vectors for *β* < *μ*) or not enough infected vectors for high *β*. In the endemic state, there is a critical birth rate *β*_0_ ≈ 0.018 = *μ* + *ω* where reducing *β* below *β*_0_, while still having it above 0.009, may actually increase the incidence rate. See Fig 4C.

**Table 2:** The sensitivity analysis. The sensitivity index *SI*_*y*_ of a variable *y* on a parameter *x* was calculated as $\left(\frac{x}{y}\right)\cdot \left(\frac{\partial y}{\partial x}\right)$, see for example [72]. The numbers were rounded to the three decimal places. Parameters are as specified in Table 1. The sensitivity index − 0.5 means that a 1% increase of a parameter value *x* will result in the 0.5% decrease of the variable *y*.

Parameter	$SI_{p_{HI}}$	$SI_{p_{NE}}$	$SI_{\left(p_{\text{HI}}-p_{\text{NE}}\right)}$	*SI*_*i*_
*a*_0_	0.496	0.498	−1.007	−0.502
*b*_0_	0.496	0.498	−1.007	−0.523
*α*	−0.493	−0.495	1.024	1.522
*β*	−0.495	−0.497	1.007	0.522
*μ*	−0.106	−0.106	0.243	0.121
*δ*	0.000	0.000	0.041	0.041
*σ*	0.117	0.117	−0.253	−0.133
*K*_*s*_	0.511	0.514	−0.990	−0.488
*K*_*p*_	−0.002	−0.002	−0.030	−0.026
*ω*	0.003	0.003	0.042	0.036
*C*_Chagas_	0	0.002	−0.977	−0.975
*C*_ITN_	0	−0.002	1.044	1.042

## Conclusions and discussion

In this paper, we modeled Chagas disease dynamics using the compartmental model developed in [44]. We parameterized the model based on values found in literature. We applied a game-theoretical approach, developed by [23], and determined the optimal voluntary use of insecticide treated nets (ITNs) to prevent the spread of infection through vector bites. We validated our model by predicting incidence rates that closely track the real incidence rates in Latin America, Mexico and Columbia. Our results confirm that individuals behave rationally and weigh the risks of insect bites against the cost of ITNs.

Our model gives two main predictions. We show that to completely eradicate the vector transmission through the voluntary use of ITNs, the cost of ITNs should be as low as possible. We also show that coupling ITN use with other means of vector control to decrease the vector presence in the households is very effective. On the other hand, in agreement with [39], if one cannot reduce the vector’s presence (or the vector birth rate) in the household below a critical threshold, increasing the vector presence may lead to a slightly lower incidence rate.

The use of ITNs has many advantages: it protects against multiple diseases such as malaria, leishmaniasis, and dengue [18], and it can be easily integrated into community health work [58, 76]. Compared to residual insecticide spraying, the use of ITNs does not require qualified spraying teams and it also requires considerably less insecticide [77]. Moreover, [78] showed that residual insecticide spraying was less effective than expected mainly because of moderate insecticide resistance and the limited effectiveness of selective treatment of infested sites only. The vectors can navigate past the nets; but most vectors that traversed the nets were early-stage nymphs, which are less likely to carry *T. cruzi* [79]. Furthermore, the spread of triatomine insects can be slowed down even if ITNs is used only on animal cages [79].

Our model can be extended in several ways. One can include disease related mortality which was omitted here for the sake of simplicity. A disease related mortality could cause a backward bifurcation and an existence of endemic equilibria even for ${ℛ}_{0}<1$ [80, 81]. One can also relax the assumption about vector migration and allow the vectors to migrate from the settlement. Finally, one can consider transmission other than between vectors and humans. Yet the findings of our simple model agree with more complex models such as [11, 39, 42, 50] which found that the best way to decrease risk of human infection is by decreasing the number of triatomine in a given area and reducing the number of domestic animals.

Although every math model has many limitations, these models can help us to understand diseases and implications of various control measures. We hope that this model helps to serve as a tool in showing the importance of ITN use to prevent Chagas disease and to minimize the domestic transmission in Latin America as stated by the London Declaration.

## Supporting information

S1 Matlab Code The matlab code used to generate the figures is available in S1 Matlab Code. (M)
